# Automated phenotyping of mammary gland tissues using computer vision systems

**DOI:** 10.3168/jds.2025-28114

**Published:** 2026-03-17

**Authors:** E. Casella, G. L. Menezes, A. L. Vang, W. S. Frizzarini, T. Cunha, G. J. M. Rosa, L. L. Hernandez, J. R. R. Dórea

**Affiliations:** 1 Department of Animal Science, The Pennsylvania State University, State College, United States, 16802; 2 Department of Animal and Dairy Sciences, University of Wisconsin, Madison, United States, 53706; 3 Department of Biological Systems Engineering, University of Wisconsin, Madison, United States, 53706

**Keywords:** Computer vision, histological phenotyping, mammary gland

## Abstract

Quantifying mammary gland tissue composition is crucial for understanding growth patterns and future lactation potential in dairy heifers, yet conventional histological analysis is labor-intensive and time-consuming. This study developed an automated phenotyping framework using deep learning-based semantic segmentation to classify tissues in mammary gland biopsies collected from Holstein heifers at 10, 26, 39, and 52 weeks of age. A data set of 130 histological images was manually annotated and divided into 383,650 patches of 448 × 448 pixels to accommodate the architecture requirements of pre-trained models. U-Net and U-Net++ models with ResNet34 encoders were trained using focal loss and evaluated through Intersection over Union (IoU) metrics. The best-performing model was a U-Net++ without attention module, achieving high IoU values across validation and test sets. Mean average percentage error remained below 5% for most tissue-time combinations, with ducts demonstrating the highest prediction accuracy despite their limited presence in early developmental stages. Adipose tissue showed the most dynamic growth pattern, increasing from approximately 2.5% of whole tissue at 10 weeks of age to over 30% at 26 and 39 weeks of age before declining to below 25% at 52 weeks of age. Strong correlations between predicted and observed pixel counts confirmed model reliability across tissues and time points. This automated approach provides a scalable, reproducible solution for histological phenotyping, enabling high-throughput quantification of mammary tissue development that was previously impractical with manual methods. The framework successfully bridges the gap between detailed tissue characterization and the demands of large-scale phenotyping studies in dairy science.

## INTRODUCTION

Tissue growth and composition are fundamental metrics in animal science, as they reflect physiological development and influence key outcomes such as productivity, health, and efficiency. Quantifying the relative abundance and organization of different tissue compartments provides insight into growth patterns, nutrient utilization, and long-term performance potential (Daniels et al., 2009; Meyer et al., 2006). Histological biopsies are the standard approach to obtain high-resolution data on tissue structure, offering precise insights into cellular structure, adipose development, and stromal remodeling. However, analyzing the images from those biopsies remains a labor-intensive and time-consuming process. Conventional workflows typically involve manual annotation and measurement of histological structures using open-source or commercial image-analysis software, often requiring hours to quantify a single histological image (Vang et al., 2023). This approach yields only a limited number of measurements, is susceptible to operator subjectivity, and constrains the scale of phenotyping studies. In addition, variability in thresholding criteria and differences between annotators can lead to inconsistencies across laboratories, further limiting reproducibility (Gurcan et al., 2009).

The ability to quantify mammary gland development at scale is especially important given the dynamic changes occurring during the prepubertal and peripubertal periods. Histological studies have demonstrated that body weight, nutrition, and milk replacer formulation significantly influence mammary parenchyma growth and fat pad composition in heifers (Capuco et al., 1997; Daniels et al., 2009). Non-invasive imaging modalities, such as ultrasound, have been explored to monitor mammary gland growth, but they lack the resolution to distinguish multiple tissue compartments reliably and provide only gross measures of tissue area (Vang et al., 2024). Thus, histological biopsies remain essential for characterizing fine tissue architecture and compartmental proportions.

Advances in computer vision and deep learning now provide an opportunity to overcome the limitations of manual histological analysis. Semantic segmentation in particular is a powerful technique in which each pixel of an image is assigned to a specific class (Asgari Taghanaki et al., 2021). Deep learning architectures such as U-Net have been widely adopted for semantic segmentation in biomedical research, demonstrating robust performance in identifying the boundaries of histological structures across multiple tissue types (Litjens et al., 2017; Ronneberger et al., 2015). More broadly, convolutional neural networks have shown strong performance in segmenting glandular and adipose tissues, including mammary gland sections in humans and other species, supporting their relevance for dairy cows’ histology (Campanella et al., 2019; Moen et al., 2019). When applied to histological microscopic images, these methods can instantly isolate epithelial, stromal, and adipose regions, enabling reproducible, large-scale phenotyping. Importantly, automated segmentation not only allows pixel-level classification but also supports geometric and morphometric analyses, including measurements of compartmental size distributions and relative proportions, which are critical for understanding developmental and physiological processes. However, to the best of our knowledge, this approach has not yet been utilized or validated for mammary histology images in dairy cows.

Therefore, in this study, we aim to develop a patch-based multilabel segmentation framework using pretrained U-Net and Unet++ models to classify tissues in histological biopsies collected from heifers at 10, 26, 39, and 52 weeks of age. By dividing whole-slide images into smaller patches, we leverage the capacity of deep learning models “pre-trained” on low-resolution images while preserving accuracy across large, high-resolution images. This approach provides a scalable and reproducible solution for histological quantification, bridging the gap between detailed tissue characterization and the high-throughput demands of modern animal science.

## MATERIALS AND METHODS

### Data collection

All procedures were approved by the Animal Use and Care Committee of the University of Wisconsin – Madison (A006270-R01). Thirty-six female Holstein calves (40 ± 5.42 kg) were born at the Blaine Dairy Cattle Center, Arlington, Wisconsin. Calves were paired by birthweight and placed on 1 of 2 treatments intended to create mammary gland growth differences (Vang et al., 2023). Average daily gain (ADG) for the high (H) nutrition value diet (Cow’s Match ColdFront Protein Blend, Land O Lakes; 27% CP, 20% Fat) animals was 0.584 ± 0.036 kg from birth through 10 weeks of age, and 0.565 ± 0.030 kg for low (L) nutrition value diet (Herd Maker Protein Blend, Land O Lakes; 22% CP, 15% Fat) animals. While the diets differed in nutrient density, the lack of significant difference in ADG during the 10-week treatment period may be attributed to increased feeding volume provided to the L group during winter months to meet maintenance requirements. Additionally, puberty occurred at 11.0 ± 0.131 mo in the H animals and 11.2 ± 0.132 in the L. Overall puberty occurred at 11.13 ± 0.186 mo. Puberty was attained when progesterone levels reached 1ng/mL for 2 consecutive weeks. Seven of the animals did not reach puberty by 12 mo of age while the rest ranged from 9.13 to 12 mo of age. Consistent with these growth and developmental patterns, Vang et al. (2023) reported no significant differences in mammary histology between treatment groups.

### Tissue collection

Mammary gland biopsies were performed on heifers at 10, 26, 39, and 52 weeks of age. Biopsies were performed at ~10 weeks on 30 out of the 36 animals as the glands of 6 animals were determined to be too small to biopsy. Additionally, not all tissue samples contained ductal structures. We had initially tried taking biopsy samples around 9 weeks of age but decided that we would give the animals another week or so to grow the mammary gland further (especially the low diet animals). Biopsy dates were scheduled at 70 +/− 3d and the average biopsy age was 69.76 d or 9.96 weeks of age. The 10 and 39 week biopsies were performed on the left rear quarter and the 26 and 52 week biopsies on the right rear quarter. The biopsies were performed by creating a 1 to 2 cm slit through the skin with a scalpel, and the capsule was dissected to take tissue cores with Integra Miltex disposable biopsy punches. At 10 weeks the 2 mm punch was used, while at 26, 39, and 52 weeks of age the 6 mm punch was used. Tissue sizes varied based on animal and age. The tissue cores collected were approximately 2 mm by 5 mm at 10 weeks of age, 6 mm by 1 to 2 cm at 26 weeks of age and beyond. Tissue was rinsed with saline and fixed for 24 h in 10% buffered formalin and then transferred to 70% ethanol. The samples were then sent to the University of Wisconsin – Madison Veterinary School of Medicine for embedding, sectioning, and staining with hematoxylin and eosin.

### Image data set

As a result of performing 30 biopsies at 10 weeks of age, and 36 biopsies at 26, 39, and 52 weeks of age, the data set consisted of 138 microscopic images. The size of microscopic images varied proportionally to tissue size, from a minimum resolution of 5,376 by 3,648 pixels to a maximum resolution of 56,640 by 59,712 pixels, and an average resolution of 25,000 by 22,000 pixels. Each pixel of the image was annotated using QuPath, an open-source software for digital pathology image analysis that labels pixels by drawing the boundaries of their respective tissue (Bankhead et al., 2017). Annotations were used to classify each pixel of the image using 4 labels, namely, whole tissue, ducts, adipose tissue, and background. Each pixel can be annotated with more than one label. For instance, a single pixel annotated as “ducts” would also be annotated as “whole tissue,” practically holding 2 concurrent outputs. A total of 130 out of 138 images were annotated, specifically 30 biopsies at 10 weeks of age, 35 biopsies at 26 weeks of age, 36 biopsies at 39 weeks of age, and 29 biopsies at 52 weeks of age. The remaining unannotated images were ignored as they did not contain any ducts.

### Data pre-processing

Training a computer vision model using the histological images at full resolution is not a feasible solution. Commonly used deep neural networks for computer vision tasks (Oliveira et al., 2021) are designed for low image resolutions, such as 224 by 224 (Simonyan and Zisserman, 2015), 448 by 448 (Redmon et al., 2016), or 572 by 572 (Ronneberger et al., 2015). That is because such computer vision models are generally “pre-trained” on large data sets of images with similar resolution, such as ImageNet (Deng et al., 2009), which contains a large collection of several every-day objects. Training the models on these large data sets improves their ability to identify relevant geometric features in the images. Then, further training on the domain-specific data set occurs with a process called “transfer learning” (Morid et al., 2021), which helps the model adapt its abilities for the specific domain-oriented task. Because these large data sets, such as ImageNet, have very low image resolution, the microscopic images would need to be rescaled to a resolution over 50 times lower than the original one, which would significantly lose details. Therefore, a patching strategy was implemented to create a mosaic of low-resolution patches that would be individually fed to the computer vision model, ultimately retaining all details of the microscopic images.

### Patching strategy

The patch size was determined based on the deep learning (DL) models adopted for our multilabel semantic segmentation task, which were: first, a U-Net (Ronneberger et al., 2015) with a pre-trained ResNet34 (He et al., 2016) encoder with an input size of 224 by 224, and second, a Unet++ (Zhou et al., 2018), with the same pretrained ResNet34 encoder. While any input size could be used, a general rule of thumb suggests using an input that is proportional to the 224 by 224 pixels used by the ResNet34 encoder (e.g., 224, 448, 672). A resolution of 448 was chosen, as it performed better than either 224 or 672, as shown in the experimental evaluation. Since all patches need to be of equal size, the original microscopic images were automatically padded with black pixels in both height and width, which consists of artificially adding black pixels to the image so that both height and width would be multiples of 448. This is basically equivalent to extending the empty background of the microscopic images, and ensures that the system does not incur into errors if the dimensions of the patches on the right and bottom edge of the figure were not of resolution 448 by 448.

To create the patches, a sliding window of size 448 by 448 pixels was moved over each image, with no overlap between consecutive patches. A single image of size H by W would therefore be transformed into several images (i.e., patches) of 448 by 448 pixels. Using common computer vision nomenclature, an image of size (1, H, W, 3) would be transformed into a vector of size (P, 448, 448, 3). Informally, a single image of height H and width W with 3 channels (one per tissue) results in P patches of height and width 448 by 448, and the same 3 channels. For instance, a histological image of resolution 56,448 by 36,736 would be transformed into a vector of size (10,332, 448, 448, 3), i.e., 10,332 patches of 448 by 448 pixels for each channel. After performing the patching strategy on each of the 138 segmented microscopic images, the original data set of 130 images was transformed into a data set of 383,650 patches. This number was calculated after discarding 85% of the patches that only contained blank background pixels with no tissue, since they would not carry any valuable information. A visual representation of the patching strategy is shown in Figure 1.

### Processing annotations

Annotations were provided by QuPath as 3 separate files for whole tissue, ducts, and adipose tissue. The annotation file of each tissue consisted of a binary file, with a value of 1 in the position of a pixel where the tissue was identified, and 0 otherwise. By stacking the 3 annotation files into one single image with 3 channels, the annotations were effectively turned into an RGB image that would show each tissue as red (R), green (G), or blue (B). The fourth label, i.e., the background, was simply considered as the set of pixels with no ducts, adipose, or whole tissue, corresponding to a value of 0 across all 3 channels of the annotation file. As shown in Figure 2, since the whole tissue file stored in the blue channel may overlap with ducts and adipose tissue, the ducts that were originally stored in the green channel are displayed as yellow, while the adipose tissue stored in the blue channel is displayed as purple. Background pixels are simple black.

### Data split methodology

To create training, validation, and testing sets, the data split methodology was based on splitting patches by animal IDs. Of the 36 animals, only 29 had full data across the 4 data collection time points. To ensure the model would be trained properly, 22 of the 29 animal IDs with full data were randomly selected and assigned to the training test, representing about 75% of the animals. Of the remaining 14 animals, 7 IDs were randomly picked and assigned to the validation set, while the remaining 7 IDs were assigned to the testing set. This corresponded to a training set of 88 images (300,000 patches, about 78% of the total), a validation set of 20 images (39,000 patches, about 10% of the total), and finally a testing set of 22 images (44,000 patches, about 12% of the total).

### Multilabel segmentation model and fine-tuning

The multilabel segmentation framework was set up using U-Net (Ronneberger et al., 2015) and U-Net++ (Zhou et al., 2018) model architectures. While Unet++ was published as an improvement over the original U-net, testing both models was a valid choice since smaller and simpler models may sometimes learn better and provide better performance. U-net is a fully convolutional neural network that was developed to excel specifically in semantic segmentation for medical imaging. It comprises an encoder and a decoder, linked by skip connections. The encoder extracts features at varying spatial resolutions with skip connections. The decoder blocks then leverage the skip connections to generate a precise segmentation mask. For the encoder, weights were taken from a ResNet34 that was pre-trained on the ImageNet data set. Two versions of the decoder were also tested, one with a spatial and channel squeeze-and-excitation (scSE) attention module (Roy et al., 2019), and one without an attention module. Several other parameters were extensively tested, and are listed in the following: patch resolutions of 224 by 224, 448 by 448, and 672 by 672 pixels; various loss functions including Cross-Entropy Loss, Dice Loss, and Focal Loss (Lin et al., 2017) with varying α (α) and γ (gamma) parameters; optimization strategies such as Stochastic Gradient Descent (SGD, with momentum) and Adam, with learning rates tuned accordingly between 0.00001 and 0.1; learning rate schedulers such as step-wise learning rate decay, and learning rate reduction when hitting a plateau; reduction factors for the schedulers were between 0.25 and 0.75, and the minimum learning rate threshold was set to 0.000001.

Since training with all data for just one epoch required several hours, most of the preliminary hyperparameter tuning was carried out on a smaller portion of 20% of the data set. Despite having a large data set, as described above, augmentation techniques were adopted to further enhance the model capabilities. A variety of approaches were considered, namely, elastic and grid geometric transformations, color-based transformations such as random brightness contrast, RGB shift, and hue saturation. However, such augmentation approaches did not seem to contribute to performance improvement and were discarded. A notable contribution was instead provided by vertical and horizontal flips, which mirror the image vertically and horizontally, as well as a 90-degree random rotation. For each batch, one of these augmentation techniques was applied with a probability of 0.5. Albumentations (Buslaev et al., 2020) was the PyTorch-based software package used for data augmentation.

Additional preliminary results for the hyperparameter tuning showed that focal loss was the best loss function, which was expected since previous reports show its ability to handle imbalanced class scenarios, as well as its ability to down-weigh the contribution of easy examples and focus more on hard examples (Jadon, 2020; Lin et al., 2017), which is a characteristics of the data set utilized in this approach. In particular, the poor presence of ducts in the early stages of mammary gland growth is an example of imbalanced classes, while easy and hard examples are represented, respectively, by the smooth and round shape of the whole tissue, as opposed to the more complex and much smaller surface of ducts. Other parameters for the loss function include no prior probability of having a positive value in the target, 2.0 was the power factor for dampening weight (also known as focal strength), and no normalization of focal loss or reduced focal loss were adopted. The optimizer adopted to adapt the weights of the model and thus minimize the loss function was the Stochastic Gradient Descent (SGD), with a learning rate reduction when hitting a plateau starting at 0.1 with a patience of 5 and momentum of 0.9. The training was done with a batch size of 32.

Having identified the primary parameters that provided the best performance, the focus switched to additional experimental evaluation to identify the performance of the best model between U-Net and Unet++, the use of an scSE attention module, and the α parameter of the loss function. For this task, the number of epochs was set to 20, as it was observed that the model no longer improved by adding more epochs.

### Experiments and evaluation metrics

First, since training with all data for just one epoch required several hours, most of the hyperparameter tuning was carried out on a smaller portion with 20% of the data set. The preliminary results on this are only discussed with the objective of introducing the major set of final parameters used in the rest of the experiments, where the intention was to focus on the results obtained by the best model, and how the model would perform in its prospective use for biomedical imaging in animal research. A more comprehensive set of results shows the hyperparameter tuning of the 3 most important parameters: 1) the segmentation model, namely, Unet and U-net++; 2) the attention module, namely, scSE and no module; and 3) the α parameter of the Focal loss function. Evaluation metrics for this experiment were focal loss and learning rate on the training set, while the Intersection over Union (IoU) is displayed for validation and testing sets. IoU is the primary metric generally adopted in the context of segmentation models. Its definition is based on the overlap of predicted and observed masks divided by their union, expressed as:

$$IoU=\frac{\left|A\cap B\right|}{\left|A\cup B\right|},$$

where A is the predicted mask, B is the observed mask, ∩ is the mathematical symbol for intersection, and ∪ is the symbol for union.

In the second experiment, using the best model obtained in the previous step, the number of observed and predicted pixels were obtained, and their percentage with respect to the whole tissue was reported. Using these percentages provides a better physiological understanding than just the mere number of pixels. In fact, the number of pixels may vary drastically based on the different image sizes contained in the data set. In the third experiment, the mean average percentage error (MAPE) between the predicted and observed pixels was evaluated for each tissue and time point. Finally, in the fourth experiment, a cross-correlation matrix of the predicted and observed values was calculated.

## RESULTS

### Baseline model evaluation

All models were trained and evaluated on The Pennsylvania State University Institute for Computational and Data Sciences (ICDS) Roar high-performance computing system, using an allocation equipped with 48 Intel^®^ Xeon^®^ Cascade Lake Gold 6248R CPU cores, one NVIDIA A100 GPU, and 384 GB of RAM. The implementation was based on PyTorch 2.6.0 with CUDA 12.8, running on Python 3.12.9. The U-Net and Unet++ models were imported from the Segmentation Models PyTorch library.

### Model evaluation

Figures 3–5 show the Intersection over Union (IoU) results for training, validation, and testing sets, respectively. The performance of the training set, displayed in Figure 3, show that each of the 8 models is capable of steadily improving performance over the 20 epochs. All models perform with an IoU over 0.8 by the second epoch and quickly converging around 0.85 for most models by the twentieth epoch. The Unet++ models with attention module seem to perform better than the others, which can be justified by the fact that they are simply more complex model which seem to be able to learn slightly better than others, although the improvement is negligible. This pattern seems to change in Figure 6, which shows the performance of the same models on the validation set. It is immediately noticeable that performance do not converge as smoothly or consistently. The cumulative trend still shows values starting just over 0.8 of IoU in the first couple epochs and then converges to about 0.85 for most models. However, the highest performance was obtained by the 18th epoch of the Unet++ model with no attention module and α parameter set to 0. While this may seem like a contradiction given the performance of the models in the training set, guidelines do recommend picking the best model based on the performance of the validation set, which are then further evaluated in the testing set. In Figure 5, the performance of the different models does follow a similar trend throughout all model configurations. However, the range of IoU is a bit smaller, ranging from the usual 0.8 to a lower value of around 0.83 in the latest epochs. This time, the performance of the best model in the validation set were confirmed in the testing set as well, in which performance of 0.834 were obtained by the same Unet++ model.

Figure 6 shows the trend of the MAPE of predicted pixel counts with respect to the observed predicted pixel count. The figure shows one line per tissue (adipose, ducts, stroma) across each data collection day (10, 24, 39, 52 weeks). Overall, most data points show strong performance, with the majority showing MAPE well within 5% for all tissues. However, a peak MAPE on the second time point at 24 weeks shows worse performance for adipose and stroma tissues, at 12.2% and 8.25% MAPE, respectively. This was probably caused by the fact that tissues were more fragmented at wk 24. At 10 weeks of age, biopsies were performed with a 2mm punch, which worked well given the small size of the mammary gland parenchyma. At 24 weeks, the 2mm punch turned out to be too small, which is why later images are fragmented, so a 6mm punch was used for later stages. It seems that fragmentation of the tissues may have affected performance since the tiling of small patches may benefit from operating on larger tissues. The fact that performance on the ducts was not affected as intensely suggests that this was in fact the issue. Additionally, considering the high correlation that studies have found between ducts and milk production, this was an encouraging result.

Figures 7,8, and 9 offer an alternative perspective on the performance of this model. The figures show the individual growth of tissues with respect to the whole tissue. Each figure shows performance separately for observed and predicted pixel count. While the standard deviation of each data point exhibits high variation across animals, a pronounced trend of growth is portrayed by the adipose tissue. A quadratic trend starts at about 2.5% at the 10-week mark, surpassing 30% at 24 and 39 weeks of age, and then going back down below 25% in the last week. A similar trend is shown by the ducts, but with a less pronounced range, well within 5% and 10% on average. Finally, stroma fluctuates slightly between 50% and 60% in the first 3 time points and then drops below 45%.

The fourth and last set of results investigates the correlation of all predicted and observed pixel counts, shown in Figure 10. This not only highlights the prediction performance but also shows correlations across tissues and time points. The diagonal of the matrix seems to confirm what was observed in the results of MAPE, with slightly lower correlations at 24 weeks, and very high correlations for the ducts. Interestingly, most correlations of tissues in the last data point show moderate to strong negative correlations, which may be due to the trend inversion seen in previous figures around this time point. Adipose tissue at 24 weeks of age also confirms the worse performance observed before, showing weak correlations with all other tissues. However, overall high correlations are present throughout the largest portion of the matrix, which on one hand shows the quality of the performance, and on the other hand is justified by the implicit relationships among the tissues.

## DISCUSSION

This study successfully demonstrated that deep learning-based semantic segmentation can automate the phenotyping of mammary gland tissues from histological images, overcoming the limitations of traditional manual analysis. By implementing a patch-based approach with pre-trained U-Net and U-Net++ architectures, we developed a framework capable of accurately classifying whole tissue, ducts, adipose tissue, and background across 138 microscopic images collected from Holstein heifers at 4 developmental time points. The selection of these architectures aligns with established standards in human computational pathology, where U-Net-based models have achieved dermatologist-level accuracy in segmenting breast cancer metastases and quantifying tumor epithelium (Bejnordi et al., 2017; Janowczyk & Madabhushi, 2016). In our application, the optimal model configuration was a U-Net++ with ResNet34 encoder and focal loss function. This configuration achieved robust performance with mean average percentage errors predominantly below 5%, demonstrating reliability across the dynamic physiological changes occurring during prepubertal and peripubertal mammary development.

The automated segmentation framework not only matched the quality of manual annotations but also enabled quantitative insights into tissue composition changes over time. The model accurately captured the pronounced quadratic growth pattern of adipose tissue and the more subtle but consistent presence of ductal structures, both of which are critical indicators of mammary gland development and future lactation capacity. High correlations between predicted and observed pixel counts across tissues and time points further validated the model's performance and biological relevance. While (Meyer et al., 2006b) described mammary development in detail, the work did not demonstrate an impairment of ductal proliferation. Instead, the differences in age at harvest was the primary cause for differences in parenchymal mass. It is difficult to find studies showing a negative correlation between prepubertal parenchymal cell number (measured by DNA as a proxy) and milk production. For instance, (Tucker et al., 1973) showed a correlation between mammary nucleic acids at 5 mo and 60 d of lactation of only 0.05 to 0.27. Furthermore, (Capuco et al., 1995) and (Waldo et al., 1998) demonstrated no effect of a 52% decrease in mammary development on milk yield in the first lactation, despite the intuitive association. Nonetheless, the capabilities of the presented technology are particularly relevant given the remaining interest in the complex relationships between early-life mammary development and subsequent lactation performance in dairy cattle (Geiger et al., 2016; Soberon & Van Amburgh, 2013). Our automated approach enables researchers to quantify these morphological changes with unprecedented precision and throughput, facilitating more nuanced investigations into the developmental mechanisms underlying variation in mammary gland structure.

Beyond efficiency gains, this approach addresses key reproducibility concerns inherent to manual histological analysis by eliminating operator subjectivity and interlaboratory variability in thresholding and measurement techniques. The framework's ability to process large volumes of high-resolution images at scale makes it particularly valuable for studies investigating the effects of nutrition, genetics, or management practices on mammary development. Specifically, recent interest has focused on the role of nutrient-responsive cell populations, such as mammary stem cells, which appear to remain quiescent until pregnancy when they support significant secretory cell expansion (Capuco et al., 2002; Soberon & Van Amburgh, 2017). While the current study utilized H&E-stained sections for tissue-level segmentation, future iterations of this framework could be trained to identify specific cell types or localized stem cell niches. Although identifying these cells typically requires specialized immunohistochemistry (IHC) stains, the integration of IHC-labeled ground truth with deep learning models could eventually enable the identification of morphological markers for these cells directly in H&E images. Such a capability would provide a powerful framework for a more mechanistic understanding of how early-life nutrition influences the long-term functional capacity of the gland.

The methodology also holds significant promise for advancing our understanding of mammary gland responses to environmental stressors and disease challenges. The current framework was developed using histological images from biopsies, allowing for the microscopic quantification of tissue-level changes. Heat stress during critical developmental periods has been shown to compromise mammary structure, with late-gestation hyperthermia reducing parenchymal mass and altering epithelial cell proliferation in offspring (Dado-Senn et al., 2020), and cyclical heat stress during lactation affecting microstructural integrity of the lactating gland (Perez-Hernandez et al., 2024). By applying computer vision to these biopsies, we can achieve a level of quantitative detail, such as identifying specific shifts in epithelial density, that often exceeds the precision of manual markers. While biopsies are necessary for microscopic ground-truth data, the ultimate goal of this work is to use these high-resolution histological measurements to improve non-invasive diagnostics. As noted by (Vang et al., 2024), there is significant interest in developing ultrasound as a reliable predictor of lactation potential. Our framework provides the objective labels necessary to train machine learning models to identify corresponding structures and developmental patterns within ultrasound images. Future research, including collaborations with medical physics, will explore advanced ultrasound measurements that are not typically accessible in standard imaging. By linking microscopic histological signatures with these macroscopic ultrasound features, we can create a more robust system for predicting mammary development and functional capacity without the need for invasive sampling.

## CONCLUSIONS

In conclusion, the proposed automated phenotyping framework represents a significant advancement in histological analysis for animal science, providing researchers with a powerful, scalable tool for quantifying tissue composition. By reducing the time and labor required for histological evaluation while improving reproducibility and enabling large-scale studies, this approach has the potential to accelerate research on mammary gland development and its implications for dairy cattle productivity and welfare. Future work will expand the training library to include tissue samples from gestation, lactation, and the dry period. This will enable the framework to identify more complex structures, such as secretory alveoli, and evaluate the impacts of environmental stressors or disease states on mature gland architecture. Ultimately, this technology has the potential to accelerate research on mammary gland development and its implications for long-term dairy cattle productivity.

## Figures and Tables

**Figure 1.**
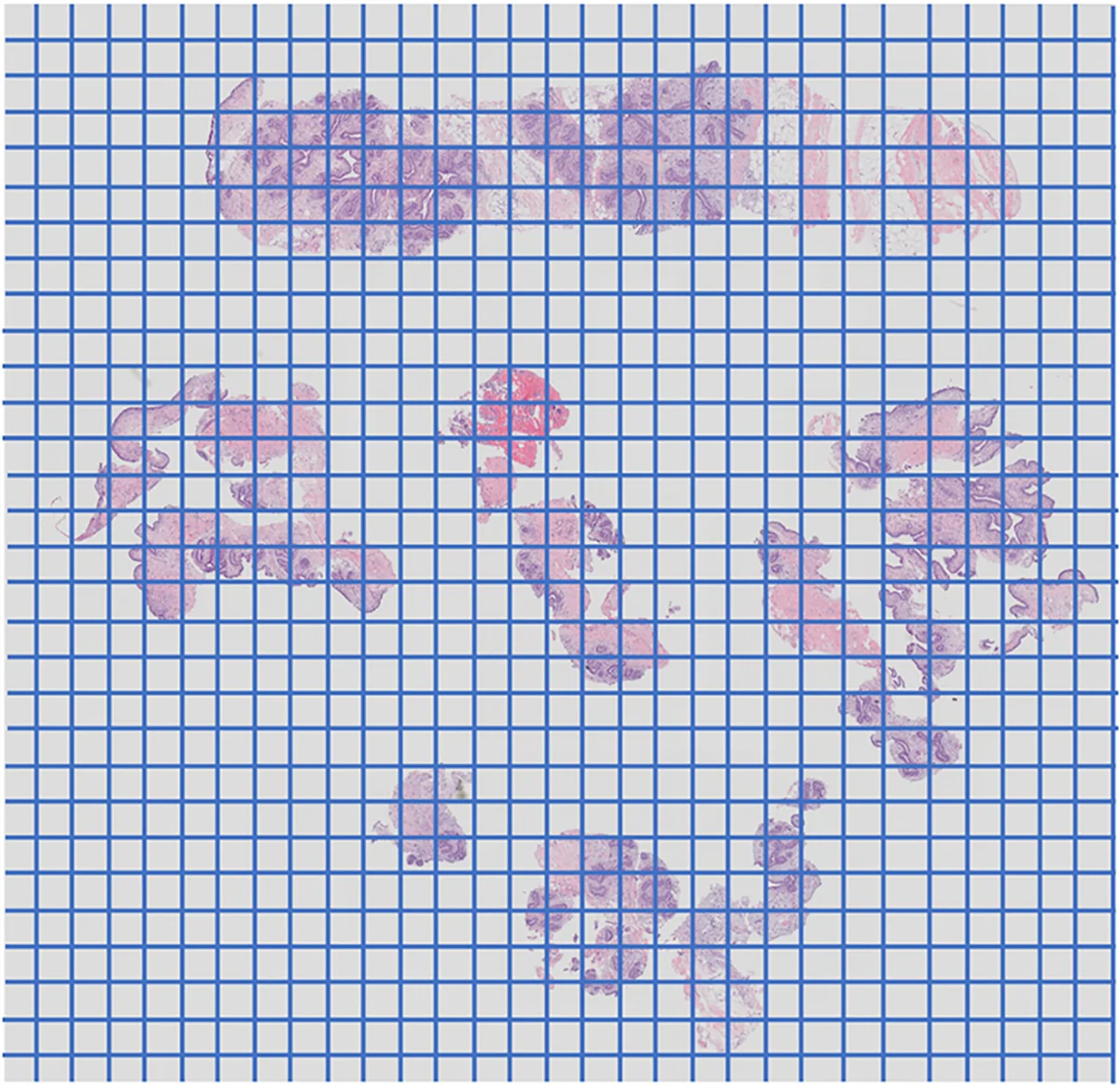
Figurative representation of the patching strategy adopted to efficiently process each microscopic image before feeding to the pretrained Unet and Unet++ models.

**Figure 2.**
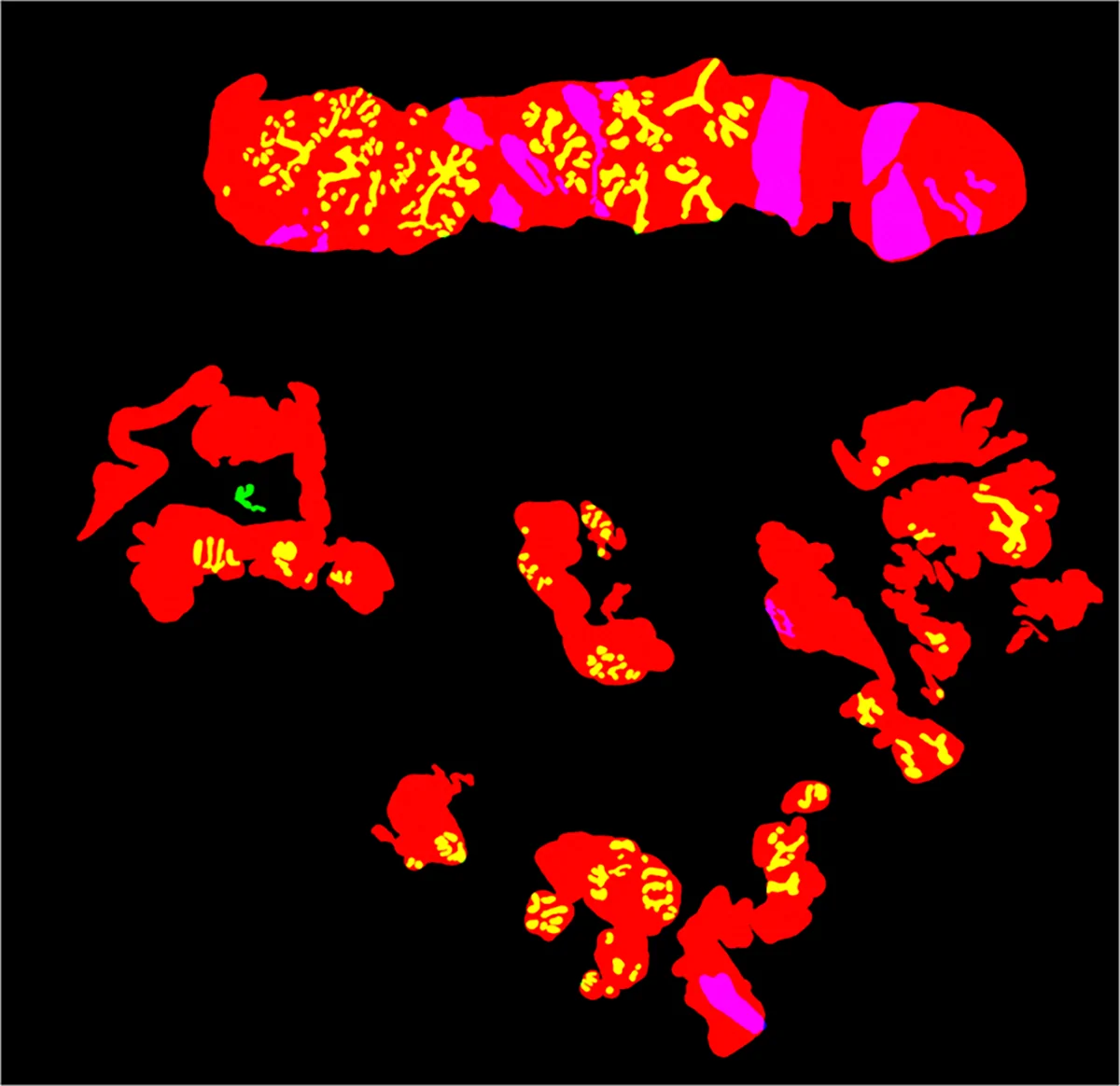
An example of an annoted histological image. Represented tissues are: whole tissue (red), ducts (yellow), and adipose tissue (magenta). Black pixels represent the background

**Figure 3.**
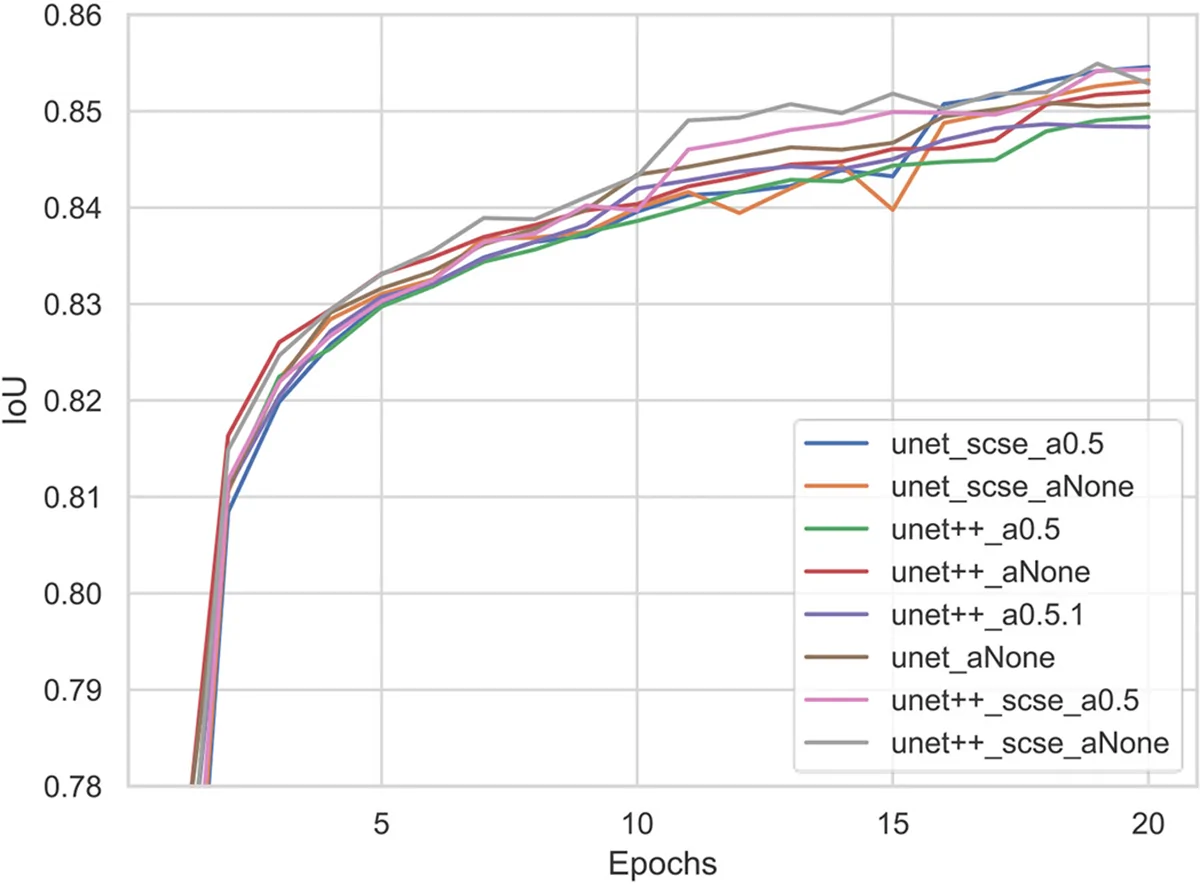
Trends of the Intersection over Union (IoU) over each epoch of the training process. Performace is shown on the training set for 8 models based on all possible combinations of: Unet and Unet++ models, using and not using an attention module, α parameter equal to 0 and 0.5.

**Figure 4.**
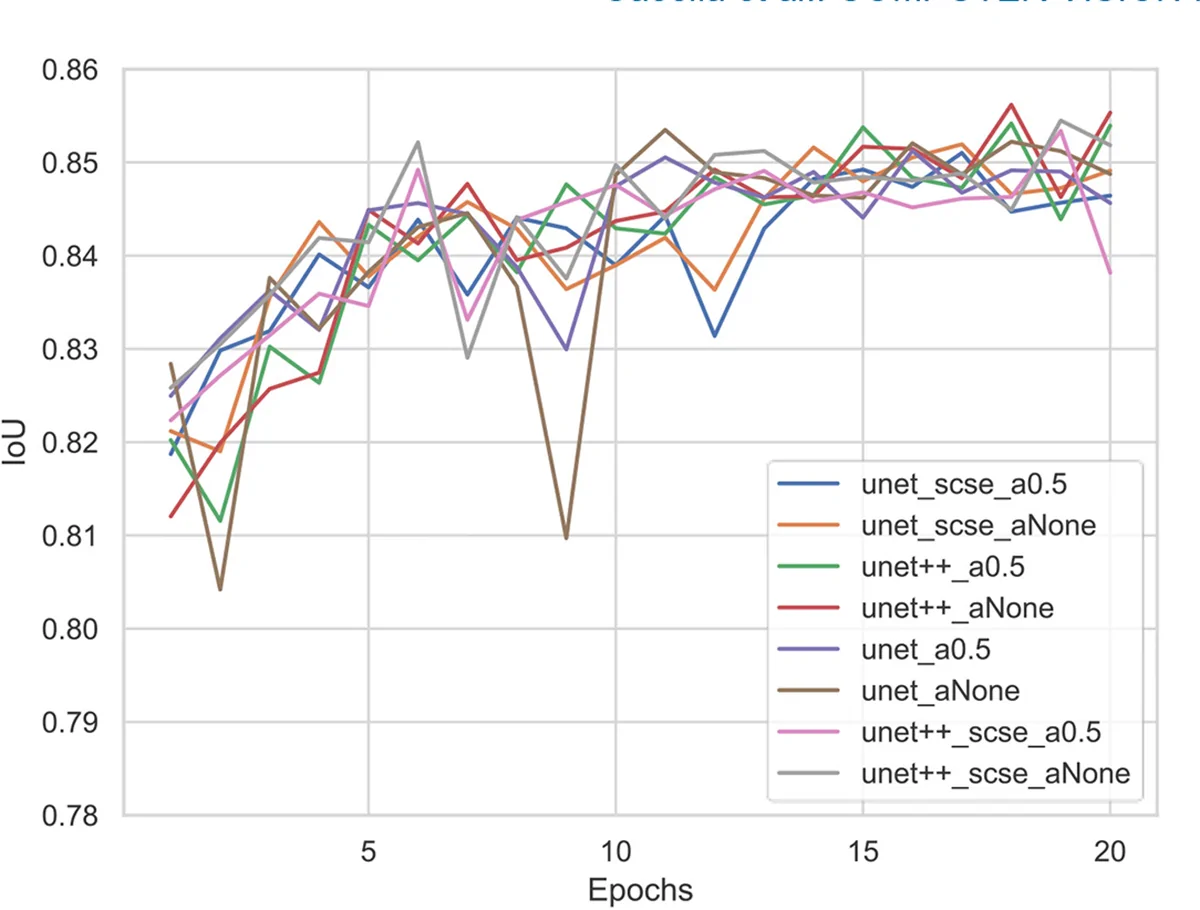
Trends of the Intersection over Union (IoU) over each epoch of the training process. Performace is shown on the training set for 8 models based on all possible combinations of: Unet and Unet++ models, using and not using an attention module, α parameter equal to 0 and 0.5.

**Figure 5.**
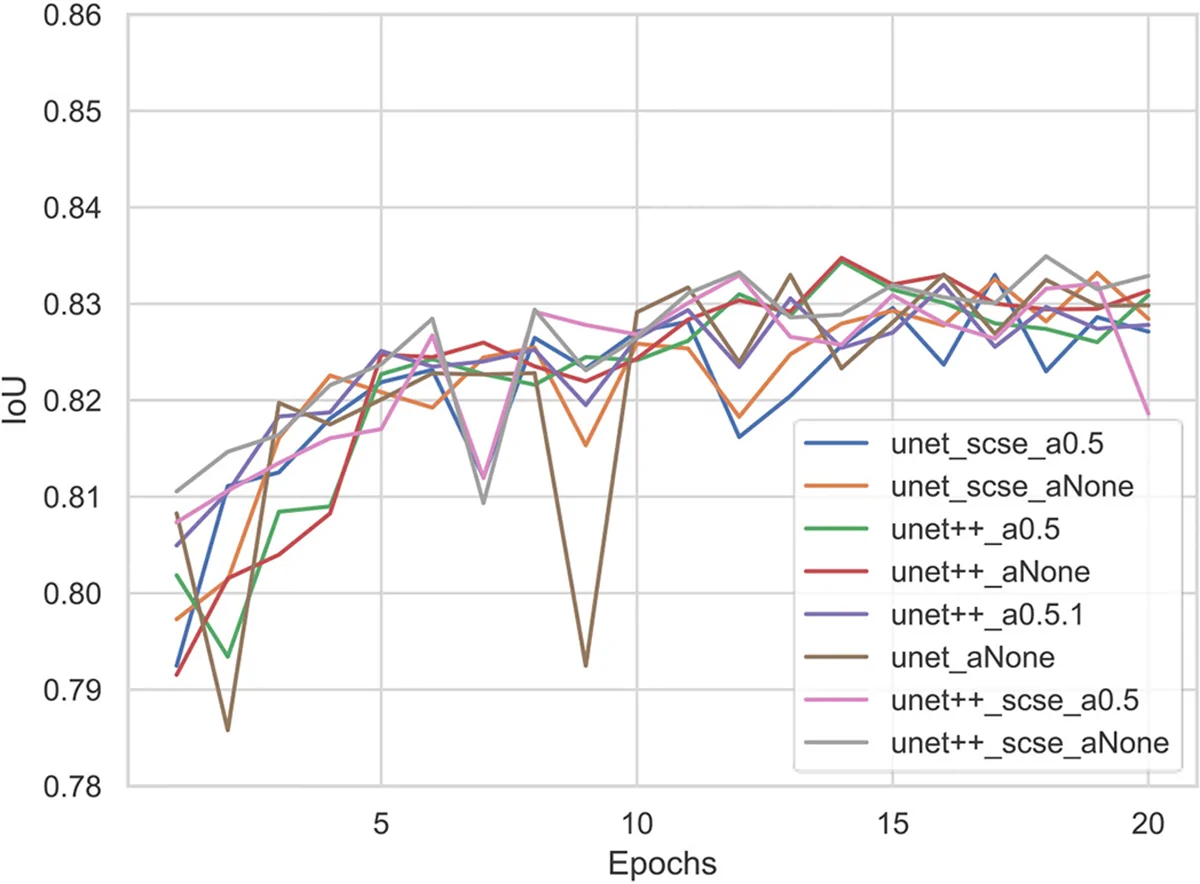
Trends of the Intersection over Union (IoU) over each epoch of the training process. Performace is shown on the training set for 8 models based on all possible combinations of: Unet and Unet++ models, using and not using an attention module, α parameter equal to 0 and 0.5.

**Figure 6.**
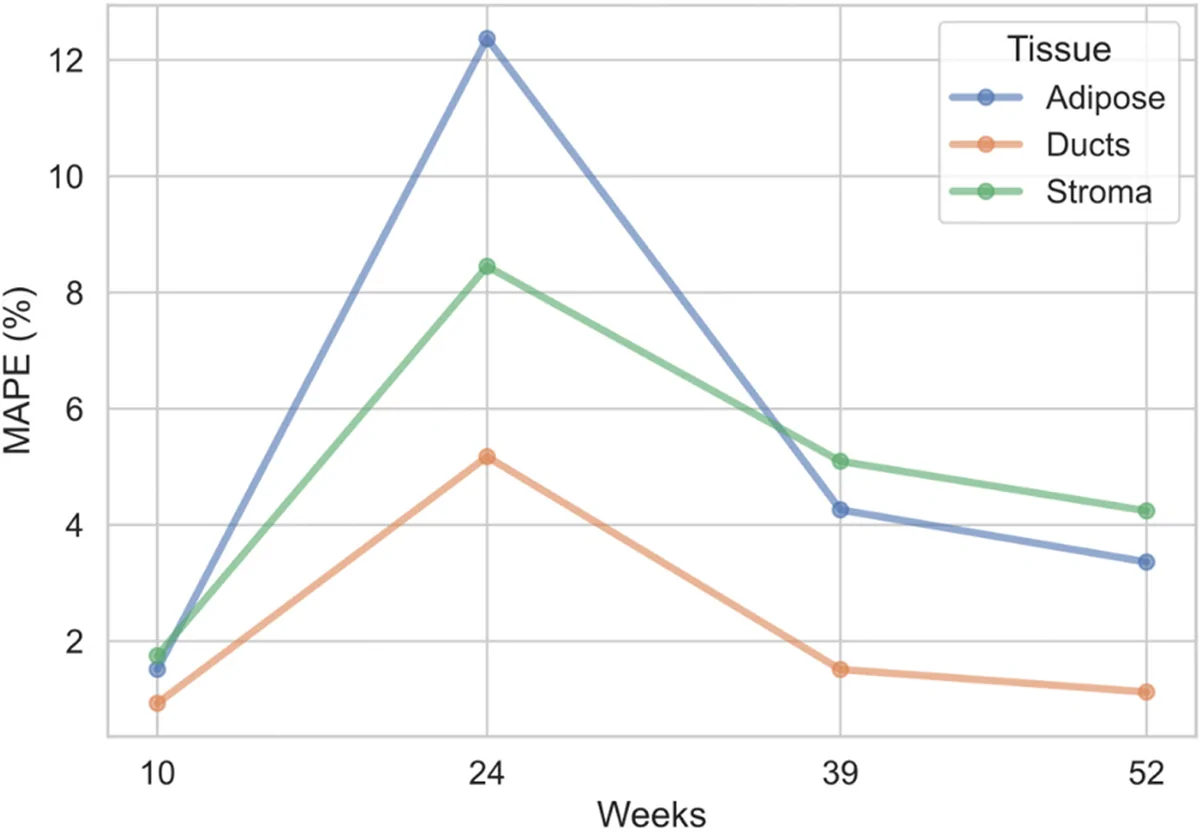
Mean average percentage error of the estimated pixel count for each tissue type over the 4 data collection periods

**Figure 7.**
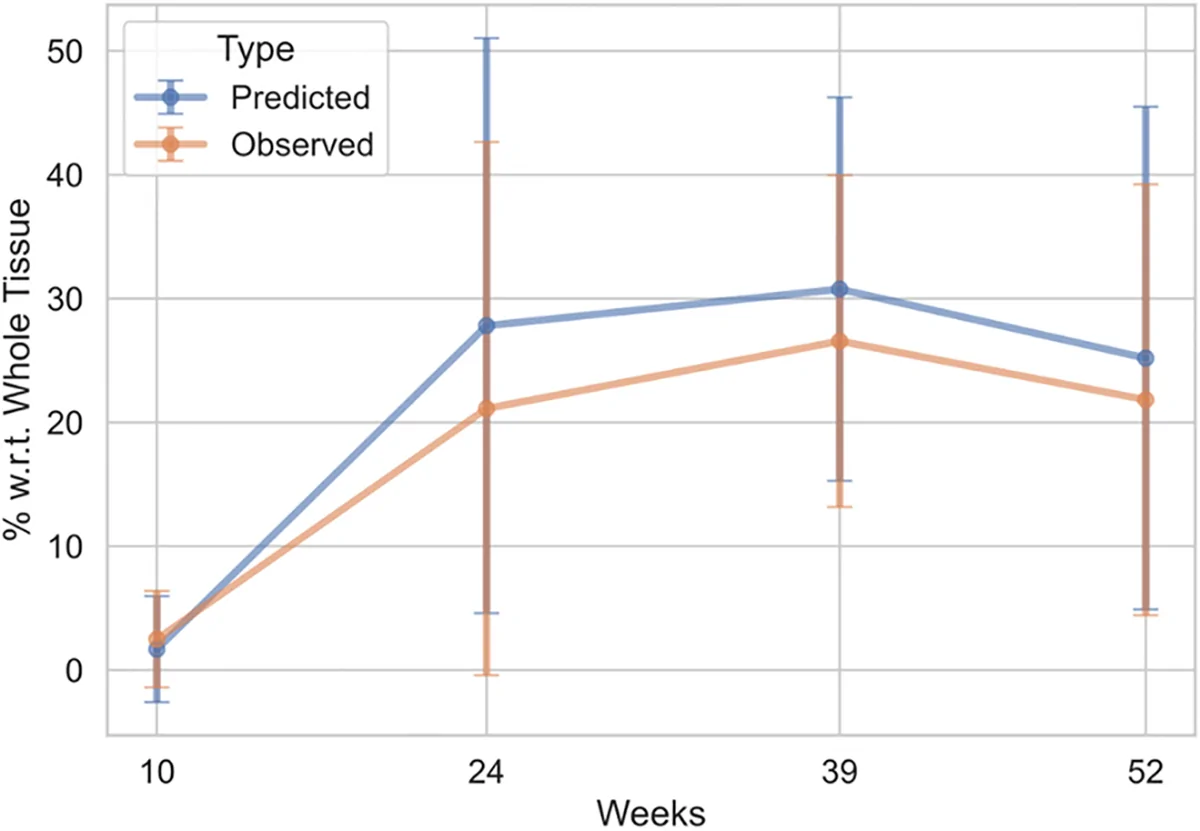
Observed and predicted percentage of adipose tissue with respect to whole tissue over the 4 data collection periods (10, 24, 39, 52 weeks).

**Figure 8.**
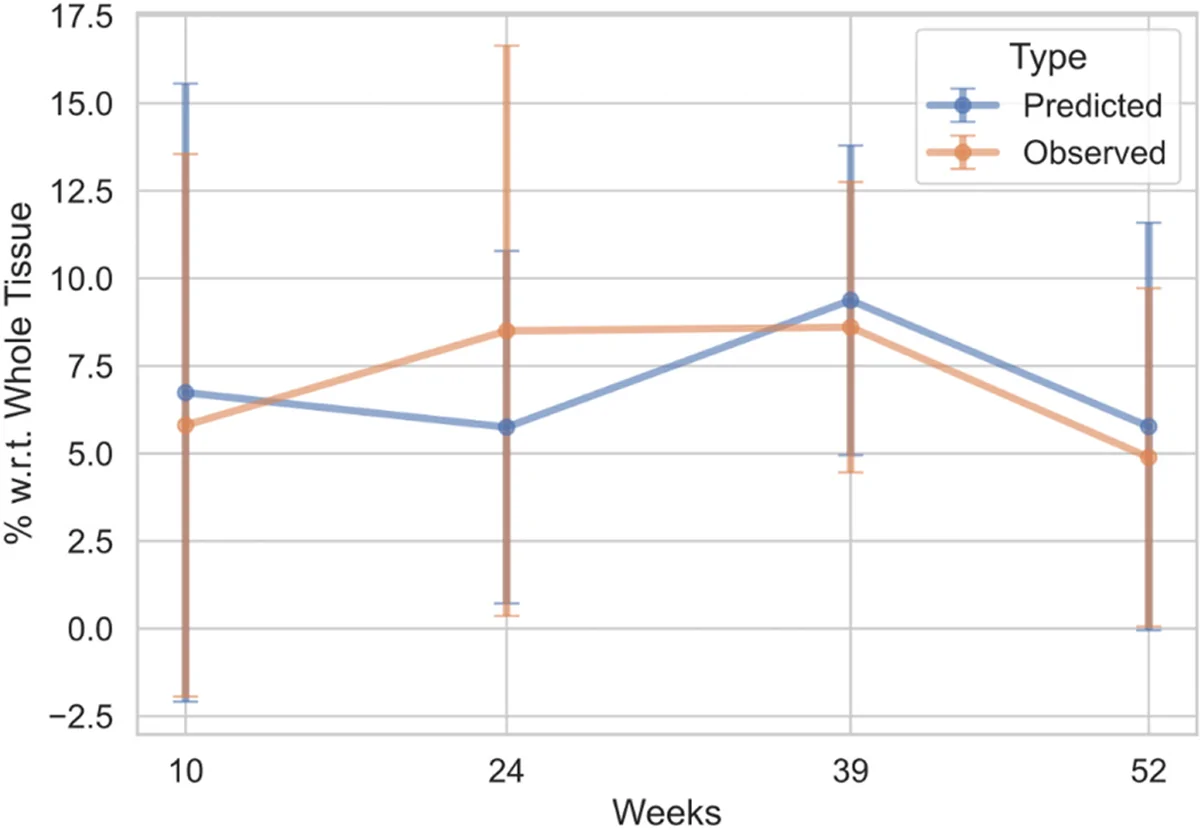
Observed and predicted percentage of ducts with respect to whole tissue over the 4 data collection periods (10, 24, 39, 52 weeks).

**Figure 9.**
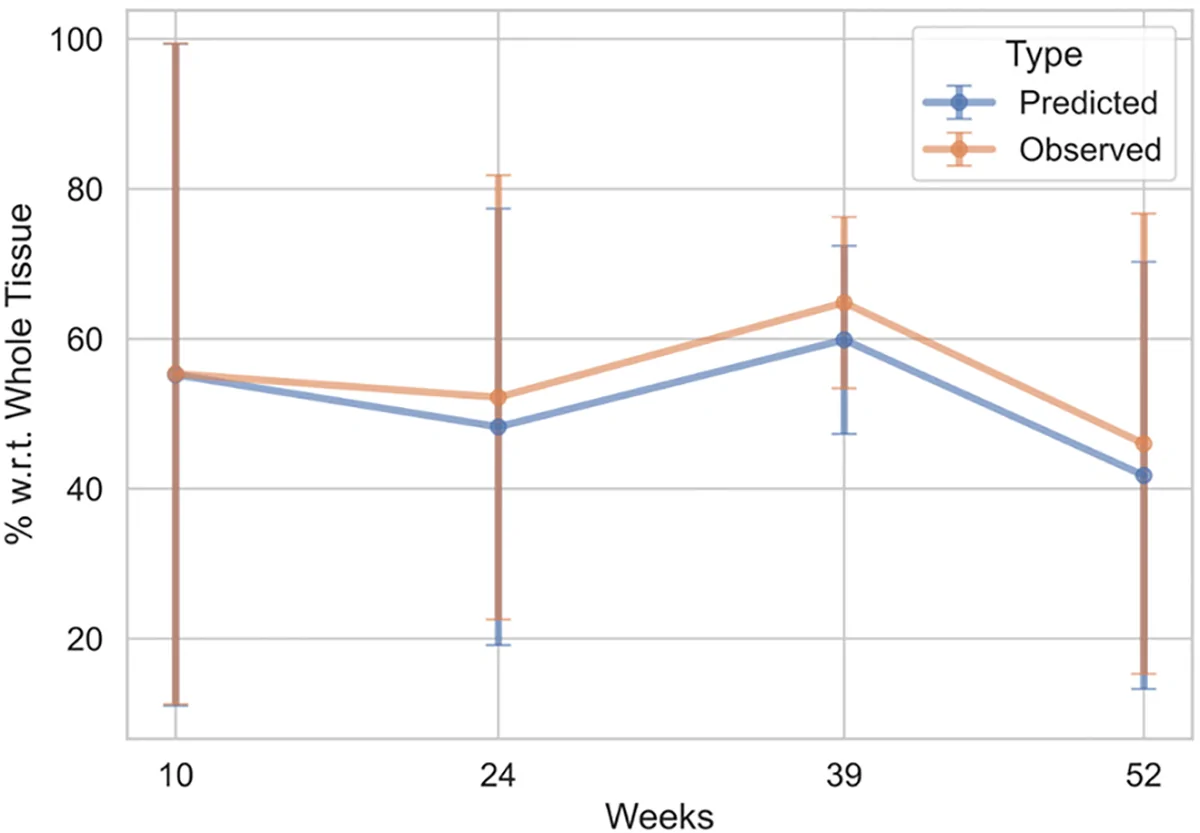
Observed and predicted percentage of stroma with respect to whole tissue over the 4 data collection periods (10, 24, 39, 52 weeks).

**Figure 10.**
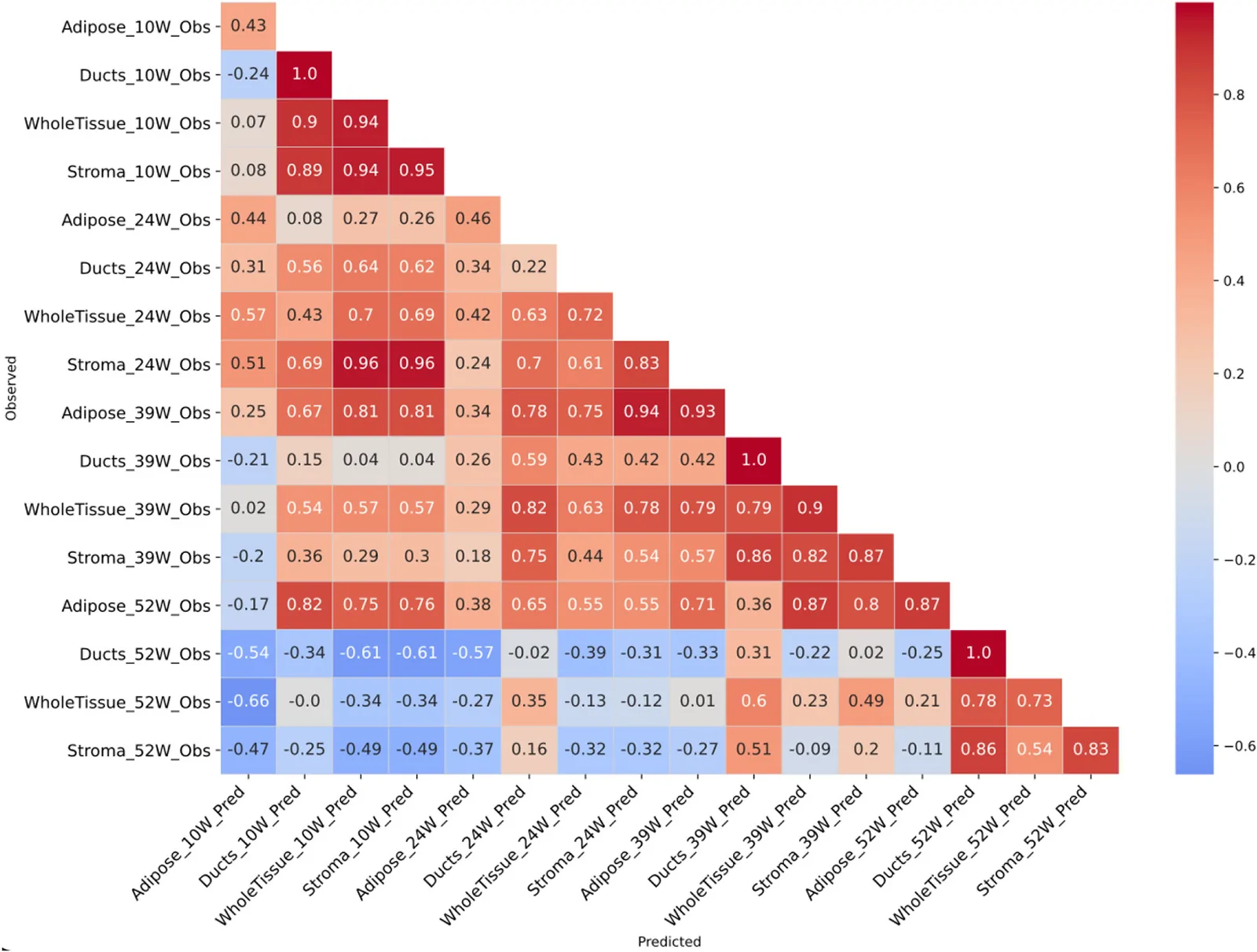
Correlation matrix between predicted and observed tissues types (adipose, whoe tissue, stroma, and adipose) at each time point (10, 24, 39, 52 weeks).
